# Effects of Virtual Reality on Adults Diagnosed with Chronic Non-Specific Low Back Pain: A Systematic Review

**DOI:** 10.3390/healthcare13111328

**Published:** 2025-06-03

**Authors:** Rocío García-de-la-Banda-García, David Cruz-Díaz, Juan Francisco García-Vázquez, María del Mar Martínez-Lentisco, Felipe León-Morillas

**Affiliations:** 1 Department of Physical Therapy, Research and Sports, FISIDEC University Center, University of Córdoba, 14940 Cabra, Spain; gbandag@hotmail.com (R.G.-d.-l.-B.-G.); jf86phythe@gmail.com (J.F.G.-V.); 2 Department of Health Sciences, Faculty of Health Sciences, University of Jaén, 23071 Jaén, Spain; fleon@ujaen.es; 3 Department of Nursing, Physiotherapy and Medicine, University of Almeria, Almería Health District, 04120 Almería, Spain

**Keywords:** chronic low back pain, low back pain, virtual reality, rehabilitation, exercise

## Abstract

Background/Objectives: Non-specific low back pain represents a high number of primary care consultations, generating a great social and economic cost. There is a higher prevalence in women, and it may be associated with multiple factors. One of the most innovative tools in rehabilitation is virtual reality-based therapy. Virtual reality positively affects the motivation of participants and generates greater adherence to treatment, so this factor could lead to long-term functional improvement. The objective of this review is to update the effectiveness of virtual reality programs on pain, disability, kinesiophobia, and changes in the thoracoabdominal musculature in patients with chronic nonspecific low back pain. Methodology: PubMed, PEDro, SCOPUS, Web of Science, and the Cochrane Library were used in this systematic review. The research question was formulated using PICOS. The Physiotherapy Evidence Database (PEDro) scale was used. Inclusion criteria were randomized clinical trials, participants were men and/or women over 18 years of age who were diagnosed with chronic non-specific low back pain, and articles that included virtual reality as a treatment. Articles with a level of evidence lower than 5/10 on the PEDro scale were excluded. Results: A total of 14 studies were included with sample sizes between 34 and 188 participants. Studies showed significant improvements in pain reduction, kinesiophobia, and disability (inflammation and motor control). Some studies showed long-term benefits, with effects maintained up to 18 months post-intervention, especially in the reduction in pain and its interference with daily activities. Conclusions: The findings of this systematic review support the efficacy of virtual reality as an effective and safe therapeutic option for the treatment of chronic non-specific low back pain.

## 1. Introduction

Low back pain is reported in 80–85% of primary care consultations related to musculoskeletal disorders, leading to a substantial burden on healthcare systems and society [1,2]. Chronic non-specific low back pain (CNSLBP) is persistent lumbar pain lasting over 12 weeks, in the absence of a specific anatomical or pathological cause. It may be accompanied by radiating or referred pain in the lower extremities [3,4]. According to the reviewed literature, it has a higher prevalence in women and can affect individuals across different age ranges, with the highest incidence observed between 41 and 50 years of age [5]. Despite being linked to various contributing factors, a definitive underlying cause is identified in only 12–15% of cases [6]. These factors include age, being overweight, general health disorders or lumbopelvic instability, occupational exposure to heavy lifting, prolonged postures and awkward postures, repetitive movements, sedentary behavior, and/or psychosocial factors [7,8,9,10,11].

Virtual reality-based therapy has emerged as one of the most innovative approaches in rehabilitation over the past decade (VRT) [12]. Virtual reality (VR) has been shown to have positive effects on participant motivation and increases treatment adherence, which may lead to long-term pain relief [13]. VRT facilitates immersive simulations that allow patients to physically interact with virtual environments replicating real-life scenarios, in a safe and real-time context [13]. Another benefit of VRT is its capacity to provide visual, auditory, or haptic feedback, enabling patients to adjust their performance in response to positive or negative stimuli, thereby enhancing motor skill learning [12,14,15]. Currently, VRT is incorporated into physical and cognitive rehabilitation programs to target functional deficits, including improvements in balance (both static and dynamic), reduction in fall-related fear and increased lower limb strength [13].

VRT of low back pain sufferers is attracting growing research interest [16,17,18,19,20,21]. Most published reviews address the effects of VRT on pain management, obtaining favorable results. In contrast, other authors state that the efficacy of VRT for chronic pain has not been demonstrated [19]. Despite the existence of evidence on this topic, the evidence is controversial [17]. Furthermore, the existing evidence shows inconclusive data on the possible effects of VRT on disability, kinesiophobia and changes in specific muscles [18,21]. For these reasons, we believe it would be interesting to conduct this literature review. Our objective in this review is to compile the most recent evidence examining the effectiveness of VRT-based programs on pain, disability, kinesiophobia and changes in the thoracoabdominal musculature in patients with CNSLBP.

## 2. Materials and Methods

### 2.1. Design and Study Registration

A systematic review of randomized controlled trials (RCTs) was carried out to determine the effects of VRT in adults diagnosed with nonspecific chronic low back pain following the “Preferred Reporting Items for Systematic Reviews and Meta-Analyses (PRISMA)” guidelines [22]. In addition, we registered the protocol for this review with the registration number CRD42024540521 in PROSPERO, the International Prospective Register of Systematic Reviews.

### 2.2. Search Strategy and Data Sources

The literature search for this systematic review was carried out between November and December 2024 by two authors (F.L-M and R.G-B) in the following databases: Pubmed, PEDro (Physiotherapy Evidence Database), SCOPUS, Web of Science (WoS) and Cochrane Library. The research question was formulated using PICOS to establish the search strategy: (1) population, older adults with chronic nonspecific low back pain; (2) intervention, VR-based therapy; (3) comparison, conventional treatment; (4) outcomes, pain, disability, kinesiophobia, and changes in the thoracoabdominal muscles; and (5) study design, randomized clinical trial (RCT). The search strategy included medical subject headings (MeSH terms) “low back pain”, “virtual reality”, and “virtual reality exposure therapy” (Table S1, Supplementary File). To conduct an ideal search process, we joined the chosen terms using Boolean operators AND and OR. Although gray literature was not included in the initial database search, additional sources such as trial registries and conference proceedings were manually reviewed when accessible. Moreover, backward and forward citation tracking (“snowballing”) was performed on key articles to identify potentially relevant studies not captured in the database search.

### 2.3. Selection of Studies and Eligibility Criteria

For the identification of potentially eligible studies, two independent reviewers (F.L-M and R.G-B) screened the titles and abstracts of all references identified by the search strategy. Inclusion criteria were randomized clinical trials involving men and/or women over 18 years of age diagnosed with chronic non-specific low back pain, and articles that included VRT for the participants and included a control group—whether a non-intervention control group, a placebo, or a comparison with conventional treatment or another treatment. However, we excluded articles with a level of evidence less than 5/10 on the PEDro scale.

### 2.4. Data Extraction and Quality Assessment

For each study chosen for this review, we collected data related to the participants (sample size, age, inclusion/exclusion criteria), the type of intervention (type of VR, frequency of sessions, number of sessions, and time elapsed), the duration of the study in each of the groups (control group and experimental group), and the results (variables, measurements, and measurement tools).

The Physiotherapy Evidence Database (PEDro) scale was used to evaluate whether the selected randomized controlled trials were scientifically sound by two researchers (F.L-M and D.C-R). The PEDro scale consists of 11 items: eligibility criteria, random allocation, concealed allocation, follow-up presence, baseline comparability, blinded subjects, blinded therapists, blinded assessors, intention-to-treat analysis, analysis between groups, and both point and variability measures. The positive response to an item corresponds to 1 point. However, the maximum score is 10 points, because the first item is excluded from this score, since it is related to external validity. A study with a score greater than 5 was considered to have a low risk of bias, a moderate risk of bias if the score was 5 or 4, and of low risk of bias if the score was 3 or lower (Table S2, Supplementary File). Risk of bias assessment will be independently performed by two unblinded reviewers, and disagreements will be resolved by consensus [23].

### 2.5. Deviation from the Protocol

No deviations from the registered protocol (PROSPERO: CRD42024540521) were made during the conduct of this systematic review. The methodology was carried out as originally planned, following PRISMA guidelines. Discrepancies in study selection were resolved by a third author (D.C-R). Inter-reviewer reliability was assessed using Cohen’s kappa coefficient, yielding a value of 0.81, which reflects strong agreement according to the Landis and Koch scale.

## 3. Results

### 3.1. Study Selection

**Figure 1:**
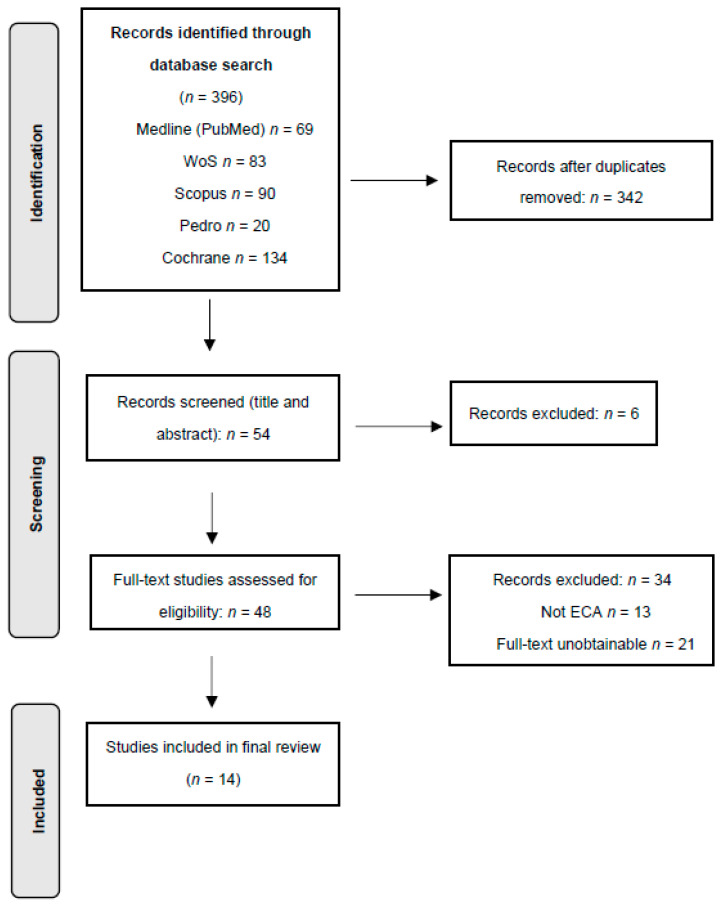
PRISMA flowchart.

The database searches resulted in 396 records. After removing duplicates and screening at title/abstract and full-text levels, 14 studies remained. Figure 1 shows a summary of the study selection process.

### 3.2. Methodological Quality

All studies met criteria 1 (specific eligibility criteria), 2 (random allocation), 4 (baseline comparability), and 10 (between-group comparisons) of the PEDro scale. In three studies, participants were blinded [24,25,26]; however, no study implemented therapist blinding. In five studies, outcome assessors were blinded [27,28,29,30,31]. Intention-to-treat analysis was performed in seven studies [24,25,26,29,32,33,34], and nine studies reported point estimates and measures of variability [27,28,29,30,31,32,35,36,37] (Table S1, Supplementary File).

Following the analysis using the PEDro scale, four studies obtained a score of 7/10 [24,25,26,29], and a total of seven studies received a score of 6/10 [27,28,31,32,33,36,37]. The mean methodological quality score of the selected studies was 6/10, indicating a moderately high level of evidence among the studies included in this review, according to the PEDro scale criteria.

### 3.3. Study Characteristics

The main characteristics of the selected studies are presented in Table 1. Among the included articles, four were conducted in the United States [25,26,33,37], four in Saudi Arabia [27,28,29,32], and one study each in Belgium [36], Pakistan [35], Turkey [31], China [30], the Netherlands [34], and the United States [24]. All studies were published in English between 2016 and 2024.

Across the studies, a total of 1281 male and female participants diagnosed with CNSLBP were included. Participant ages ranged from 18 to 85 years. The smallest sample size was reported by Li et al. [30] with 34 participants, while the largest samples were observed in the studies by García et al. [25,26] and Maddox et al. [24], each with 188 participants. Regarding dropouts, two were reported in the study by Nambi et al. [27], nine in García et al. [25], two in Nambi et al. [32], thirty-two in García et al. [26], and six in the study by Groenveld et al. [34]

All experimental group interventions involved the use of VRT [24,25,26,27,28,29,30,31,32,33,34,35,36,37]. Of the fourteen studies reviewed, five [27,28,29,30,32] compared outcomes of groups receiving different interventions. These included isokinetic exercises [27,29,32], balance exercises [27,28,29,32], motor control exercises, and thermomagnetic therapy [30]. The remaining nine studies compared VRT interventions with a single control group performing one of the following: simulated VRT [24,25,26,33], conventional physiotherapy (including heat therapy, hamstring stretching, strengthening exercises, TENS, ultrasound) [29,35], pelvic tilts [36,37], or standard care with no additional intervention [34].

The duration of intervention in the experimental group varied across studies: one study included an 18-month follow-up [24], others lasted 12 weeks [26], 8 weeks [26,33], 4 weeks [27,28,32,34,35,37], 2 weeks [30,31], 1 week [37], and one study involved a single session [36]. Regarding treatment frequency, interventions were delivered daily in some studies [24,25,26,33,34], three times per week [27,32], or five times per week [29,30,31,32]. One study involved only one session [36], and two studies did not specify the number of sessions [27,32].

The outcome variables were assessed using diverse tools and methodologies. Among the 14 studies, 13 evaluated pain [24,25,26,27,28,29,30,31,33,34,35,36,37], 3 assessed kinesiophobia [31,32,36], 5 measured disability [30,31,35,36,37], 3 investigated musculoskeletal changes in patients with CNSLBP [27,28,30], and 2 assessed quality of life [24,34]. Pain was measured using the Visual Analog Scale (VAS) in seven studies [27,29,30,31,32,34,35], the Numeric Pain Rating Scale (NPRS) in one study [36], the Defense and Veterans Pain Rating Scale (DVPRS) in four studies [24,25,26,33], and the McGill Pain Questionnaire in one study [37]. Additionally, Groenveld et al. [34] used a daily pain scale as a key variable.

Kinesiophobia was evaluated using the Tampa Scale for Kinesiophobia (TSK) in three studies [29,31,33]. Disability was assessed using the Roland–Morris Disability Questionnaire (RMDQ) in two studies [36,37] and the Oswestry Disability Index (ODI) in three studies [30,31,35]. Musculoskeletal changes were measured through MRI and ultrasound in two studies [27,28], and electromyography in one study [30].

Quality of life was measured using the SF-12 in one study [34] and the Patient-Reported Outcomes Measurement Information System (PROMIS) in another [24], adding an important dimension to the analysis of VR effects on overall patient well-being. Notably, although most studies did not evaluate long-term outcomes, the study by Maddox et al. [24] provided data on the sustained efficacy of the intervention at 18 months, thereby enriching the understanding of the potential long-term benefits of VRT in the management of CNSLBP.

No statistically significant differences were found in sociodemographic characteristics or baseline outcome measures between the study groups in any of the included articles.

The studies reported significant improvements in favor of VRT interventions across multiple domains. Positive effects were observed on inflammatory biomarkers [27,28], pain intensity and duration [24,32,37], sleep quality [25,26,32], stress reduction [25,26], physical function [33], activation time of the transversus abdominis [30], kinesiophobia [31,32,36], running and jumping performance [29], and quality of life [34].

In recent studies, Maddox et al. [24] demonstrated that VRT produced significant and long-lasting reductions in pain intensity and pain-related stress, with effects sustained up to 18 months post-treatment. Additionally, Groenveld et al. [34] reported a significant reduction in both “worst daily pain” and “least daily pain”, although no significant improvements were found in overall quality of life.

One study [27] reported significant differences in the cross-sectional area of the psoas, quadratus lumborum, multifidus, and erector spinae muscles in favor of the isokinetic exercise group compared to the VRT group. However, another study [28] observed greater transverse diameters of the same muscle groups in the VRT group compared to those receiving combined physical rehabilitation and Swiss ball balance training.

## 4. Discussion

This systematic review evaluated the effectiveness of virtual reality-based rehabilitation interventions in reducing pain, kinesiophobia and disability, as well as in improving quality of life in patients with chronic non-specific low back pain (CNSLBP). Based on the analysis of fourteen studies included in this review [24,25,26,27,28,29,30,31,32,33,34,35,36,37], virtual reality emerged as an effective therapeutic alternative comparable to conventional interventions and, in some cases, was even more effective.

From a methodological standpoint, the included studies exhibited moderate to high quality, with all scoring above 5 on the PEDro scale. No significant sociodemographic differences were observed among participants, indicating that VR may be a broadly applicable intervention. However, the absence of therapist blinding in most studies introduces a potential performance bias that should be taken into account when interpreting the results.

Findings related to musculoskeletal changes are heterogeneous. While some studies [27,32] report greater increases in muscle thickness in the VR group compared to conventional rehabilitation, others suggest that isokinetic training may result in a larger increase in the cross-sectional area of specific muscle groups [32]. These differences may be explained by variations in intervention protocols, types of devices used and treatment duration.

From a biochemical perspective, virtual reality has demonstrated a positive effect on the modulation of inflammatory biomarkers. Significant reductions have been reported in levels of C-reactive protein (CRP), tumor necrosis factor-alpha (TNF-α) and interleukins (IL-2, IL-4, IL-6), suggesting that VR training may elicit anti-inflammatory responses comparable to those induced by conventional physical exercise [27,28,38]. These findings are particularly relevant in the context of chronic pain, where inflammation plays a central role in the persistence of pain and functional impairment.

Additionally, some studies have indicated that VR enhances pain perception through mechanisms of cognitive distraction and neuroplasticity. Immersion in a virtual environment can modulate the activation of brain areas related to pain processing, such as the somatosensory cortex, the insula, and the anterior cingulate cortex [28]. These effects may contribute to a significant reduction in pain perception in chronic low back pain patients, promoting better tolerance to therapeutic exercise and greater adherence to rehabilitation programs.

At the functional level, integrating VR into conventional physiotherapy programs is associated with significant improvements in gait and postural stability. Previous studies have demonstrated that chronic low back pain patients exhibit alterations in gait speed and activation of the supplementary motor cortex during motor imagery tasks [17,39]. In this regard, VR could act as a neurocognitive facilitator, enhancing connectivity between motor areas and optimizing postural control mechanisms. However, evidence regarding its impact on balance remains inconsistent. Jo et al. [40] and Raza et al. [41] have emphasized the importance of lumbar stabilization exercises, whereas Groenveld et al. [34] concluded that VR did not produce significant improvements in postural control.

Another noteworthy finding is the reduction in the use of over-the-counter analgesics following VR treatment, although no significant changes were identified in the consumption of prescribed opioids [33]. This could indicate a potential benefit of VR in pain self-management, reducing reliance on pharmacological treatments and their associated adverse effects.

Furthermore, virtual reality has been shown to be beneficial for psychological aspects associated with chronic low back pain, such as anxiety and depression. Several studies have demonstrated that VR, by integrating interactive and controlled stimuli, can provide patients with a sense of control over their condition, reducing fear of movement and enhancing self-confidence in daily activities [31,32,36]. Additionally, VR may contribute to a reduction in pain catastrophizing, leading to less interference with patients’ quality of life.

Despite the promising findings, future research should address several important considerations. Although previous reviews and meta-analyses support the efficacy of VR in reducing pain and improving function [17,42,43], evidence regarding potential adverse effects such as motion sickness (cybersickness) resulting from discrepancies between visual input and vestibular feedback remains limited. Investigating how these side effects influence adherence and treatment outcomes is essential. Furthermore, studies should examine technological barriers encountered by older adults who may face challenges in navigating virtual environments or using VR devices. Gaining a better understanding of these limitations will contribute to the development of more accessible and user-friendly VR interventions tailored to diverse populations [39].

One significant limitation of this review is the absence of a meta-analysis. The considerable heterogeneity among the included studies (for example, in intervention protocols, durations, virtual reality systems, outcome measures, and assessment tools) prevented the execution of a meaningful pooled analysis. This methodological variability also limited the feasibility of quantitative synthesis. Future systematic reviews may address this issue by selecting more homogeneous datasets that allow for meta-analytic approaches. Although several recent meta-analyses have explored the effectiveness of virtual reality interventions for chronic low back pain [17,42,43], our study includes trials published up to 2024, broadening the scope with more recent and diverse evidence. Compared to prior analyses, this review incorporates a wider range of outcomes, populations, and intervention modalities, offering a more comprehensive synthesis of the current state of the evidence.

Finally, it is important to highlight that the heterogeneity of VR systems (ranging from non-immersive 2D screens to fully immersive head-mounted displays) likely influenced the variability of clinical outcomes. Recent studies suggest that factors such as the degree of immersion, interactivity, type of feedback (visual, auditory, haptic), and personalization of virtual environments significantly impact user engagement and therapeutic efficacy [39,44]. Therefore, future research should aim to standardize VR intervention protocols and report detailed technical specifications to enhance reproducibility and comparability across studies.

## 5. Conclusions

The findings of this systematic review support the effectiveness of VR as a therapeutic tool in the management of chronic low back pain. Its application has been shown to contribute to pain reduction, kinesiophobia, and disability, with potential effects on inflammatory modulation and motor control improvement.

Compared to other conventional treatments, such as thermomagnetic therapy, motor control exercises, or combined physical rehabilitation, VR has demonstrated comparable or superior clinical efficacy [30,35]. Additionally, its use in home-based settings could represent an accessible and sustainable long-term treatment strategy [24,33,44].

Despite these promising findings, some limitations exist in the available evidence. The heterogeneity of VR devices and protocols complicates the result generalization, and the lack of long-term follow-up studies prevents a definitive understanding of VR’s sustained impact. Future research should address these limitations through well-designed, controlled clinical trials with greater methodological rigor and standardized interventions.

In conclusion, VR emerges as an effective and safe therapeutic option for the treatment of chronic low back pain, with the potential to be integrated into conventional rehabilitation programs and improve the quality of life of affected patients.

## Figures and Tables

**Table 1:** Characteristics of the studies.

Author (Year)	Sample	Intervention	Duration	Variable	Instruments	Results
Maddox et al. (2024) [24]	EG VRT = 94, CG = 94	VRT	8 weeks	Pain, Physical Function, QoL	DVPRS, PROMIS	Significant pain reduction (*p* < 0.001). Improved physical function and QoL (effects up to 18 months).
Groenveld et al. (2023) [34]	EG VRT = 20, CG = 21	VRT	4 weeks	Pain, QoL	VAS, SF-12	Significant reduction in daily pain (*p* < 0.001). No significant changes in QoL.
Afzal et al. (2022) [35]	EG VRT = 42, GP = 42	VRT, Physiotherapy	4 weeks (12 sessions)	Pain, Disability	VAS, MODI	Significant pain and disability reduction (*p* < 0.05). Greater effects in RV.
García et al. (2022) [25]	EG VRT = 94, CG = 94	VRT	8 weeks	Pain, Physical Function, Sleep, Mood, Stress	DVPRS, DVPRS-II, PROMIS	Pain reduction (*p* = 0.001, sustained 6 months). Less interference of pain with activity, mood, and sleep (*p* < 0.001). Lower stress levels (*p* = 0.001).
García et al. (2022) [26]	EG VRT = 94, CG = 94	VRT	3 weeks	Pain, Physical Function, Sleep, Mood, Stress	DVPRS, DVPRS-II, PROMIS	Pain intensity lower in VRT (*p* = 0.0017). Significant time × treatment interaction (*p* = 0.0011).
Nambi et al. (2022) [27]	EG VRT = 19, EG IKE = 19, CG = 20	VRT, IKE, Exercise	4 weeks	Pain, Muscle CSA, Biomarkers	VAS, MRI, US, Blood Test	Pain reduction (*p* = 0.001), greatest in IKE. Increased muscle CSA, highest in IKE. Significant biomarker changes, best in VRT.
García et al. (2021) [33]	EG VRT = 89, CG = 90	VRT	8 weeks	Pain, Physical Function, Sleep, Mood, Stress	DVPRS, DVPRS-II, PGIC, PROMIS, PCS, PSEQ-2, CPAQ-8	Greater pain reduction in VRT (*p* < 0.001). Improved physical function and sleep (*p* < 0.001).
Li et al. (2021) [30]	EG VRT = 11, EG MCE = 12, CG = 11	MCE, VRT	2 weeks	Pain, Disability, Muscle Activation	VAS, ODI, sEMG	Increased muscle activation (*p* < 0.05). No significant differences in disability.
Nambi et al. (2021) [32]	EG VRT = 20, EG IKT = 20, CG = 20	VRT, IKT	4 weeks	Pain, Kinesiophobia, Stress Hormones	VAS, TSK-17, Blood Test	Pain reduction (*p* < 0.001), greater in VRT and IKT. Reduced kinesiophobia and stress hormones, highest in VRT.
Matheve et al. (2020) [36]	EG VRT = 42, CG = 42	VRT + Incline Exercises	1 session (2 × 2)	Pain, Disability, Catastrophizing, Kinesiophobia	NPRS, RMDQ, PCS, TSK	Lower pain intensity, pain catastrophizing, and kinesiophobia (*p* < 0.02). Greater effects in VRT.
Nambi et al. (2020) [29]	EG IKT = 15, EG VRT = 15, CG = 15	IKT, VRT	4 weeks	Pain, Player Welfare, Perfomance	VAS, Player Welfare Q, Sprint and Jump Tests	Pain reduction (*p* ≤ 0.001), greatest in VRT. Improved sprint and jump performance.
Nambi et al. (2020) [28]	EG VRT = 12 EG CPR =12 CG = 12	VRT	4 weeks	Muscle CSA, Biomarkers	MRI, US, Blood Test	Increased muscle CSA (*p* < 0.01). Significant biomarker changes, best in VRT.
Yilmaz Yelvar et al. (2017) [31]	EG = 22, CG = 22	VRT	2 weeks	Pain, Kinesiophobia, Disability, QoL, Function	VAS, TKS, ODI, NHP, TUG, 6MWT	Significant improvements in pain, kinesiophobia, and function (*p* < 0.01). No significant changes in ODI and NHP.
Thomas et al. (2016) [37]	EG = 26, CG = 26	VRT	Multiple sessions	Pain, Disability, Lumbar Flexion	McGill Pain Q, RMDQ	Significant pain reduction (*p* < 0.01). No changes in disability or lumbar flexion.

EG = Experimental Group; GC = Control Group; VRT = Virtual Reality Treatment; QoL = Quality of Life; DVPRS = Defense and Veterans Pain Rating Scale; PROMIS = Patient-Reported Outcomes Measurement Information System; VAS = Visual Analog Scale; MODI = Modified Oswestry Disability Index; IKE = Isokinetic Exercise; MRI = Magnetic Resonance Imaging; US = Ultrasound; CRP = C-Reactive Protein; TNF-α = Tumor Necrosis Factor Alpha; IL = Interleukins; PGIC = Patient Global Impression of Change; PCS = Pain Catastrophizing Scale; PSEQ-2 = Pain Self-Efficacy Questionnaire (2-item version); CPAQ-8 = Chronic Pain Acceptance Questionnaire (8-item version); TSK = Tampa Scale for Kinesiophobia; MCE = Motor Control Exercise; sEMG = Surface Electromyography; IKT = Isokinetic Training; RMDQ = Roland Morris Disability Questionnaire; NPRS = Numeric Pain Rating Scale; APA = Anticipatory Postural Adjustment; CPA = Compensatory Postural Adjustment; NHP = Nottingham Health Profile; ODI = Oswestry Disability Index; TUG = Timed Up and Go Test; 6MWT = 6-Minute Walk Test; SF-12 = Short Form-12 Questionnaire; McGill Pain Q = McGill Pain Questionnaire.

## Data Availability

The original contributions presented in this study are included in the article/Supplementary Material. Further inquiries can be directed to the corresponding author.

## Supplementary Materials

The following supporting information can be downloaded at: https://www.mdpi.com/article/10.3390/healthcare13111328/s1, Table S1: Search Strategy; Table S2: PEDRo Scale Bias assessment.

## Abbreviations

The following abbreviations are used in this manuscript:

APA	Anticipatory Postural Adjustment;
CNSLBP	Chronic non-specific low back pain.
CPA	Compensatory Postural Adjustment;
CPAQ-8	Chronic Pain Acceptance Questionnaire (8-item version);
CRP	C-Reactive Protein;
DVPRS	Defense and Veterans Pain Rating Scale;
EG	Experimental Group;
GC	Control Group;
IL	Interleukins;
IKE	Isokinetic Exercise;
MCE	Motor Control Exercise;
McGill Pain Q	McGill Pain Questionnaire.
MODI	Modified Oswestry Disability Index;
MRI	Magnetic Resonance Imaging;
NHP	Nottingham Health Profile;
NPRS	Numeric Pain Rating Scale;
ODI	Oswestry Disability Index;
PCS	Pain Catastrophizing Scale;
PGIC	Patient Global Impression of Change;
PROMIS	Patient-Reported Outcomes Measurement Information System;
PSEQ-2	Pain Self-Efficacy Questionnaire (2-item version);
QoL	Quality of Life;
RMDQ	Roland Morris Disability Questionnaire;
sEMG	Surface Electromyography;
SF-12	Short Form-12 Questionnaire;
TNF-α	Tumor Necrosis Factor Alpha;
TSK	Tampa Scale for Kinesiophobia;
TUG	Timed Up and Go Test;
US	Ultrasound;
VAS	Visual Analog Scale;
VR	Virtual Reality;
VRT	Virtual Reality Treatment;
6MWT	6-Minute Walk Test;
