# Supplementary material for: Effects of Virtual Reality on Adults Diagnosed with Chronic Non-Specific Low Back Pain: A Systematic Review
Source: Healthcare (Basel). 2025 Jun 3;13(11):1328. doi: 10.3390/healthcare13111328 (PMC12155277; doi:10.3390/healthcare13111328)
Supplement: Supplementary file 1 [file healthcare-13-01328-s001.zip › healthcare-3628408-supplementary.pdf]

Supplementary File 1

Table S1. The search strategy.

Database	Strategy
Pubmed	“low back pain” OR "chronic low back pain") AND ("virtual reality" OR “virtual reality exposure therapy)
PEDro, Scopus and Cochrane	“low back pain” AND “virtual reality”
WOS	“low back pain” AND “virtual reality”
	“low back pain” AND (“virtual reality” OR “virtual reality exposure therapy”

Supplementary file 2

Table S2. PEDro scale bias assessment												
Authors	1	2	3	4	5	6	7	8	9	10	11	Points
Maddox et al. (2024) [24]	YES	YES	YES	YES	YES	NO	NO	YES	YES	YES	NO	7/10
Groenveld et al. (2023) [34]	YES	YES	NO	YES	NO	NO	NO	YES	YES	YES	NO	5/10
Afzal et al. (2022) [35]	YES	YES	NO	YES	NO	NO	NO	YES	NO	YES	YES	5/10
García et al. (2022) [25]	YES	YES	YES	YES	YES	NO	NO	YES	YES	YES	NO	7/10
García et al. (2022) [26]	YES	YES	YES	YES	YES	NO	NO	YES	YES	YES	NO	7/10
Nambi et al. (2022) [27]	YES	YES	NO	YES	NO	NO	YES	YES	NO	YES	YES	6/10
García et al. (2021) [33]	YES	YES	YES	YES	NO	NO	NO	YES	YES	YES	NO	6/10
Li et al. (2021) [30]	YES	YES	NO	YES	NO	NO	YES	NO	NO	YES	YES	5/10
Nambi et al. (2021) [32]	YES	YES	NO	YES	NO	NO	NO	YES	YES	YES	YES	6/10
Matheve et al. (2020) [36]	YES	YES	YES	YES	NO	NO	NO	YES	NO	YES	YES	6/10
Nambi et al. (2020) [29]	YES	YES	NO	YES	NO	NO	YES	YES	YES	YES	YES	7/10
Nambi et al. (2020) [28]	YES	YES	NO	YES	NO	NO	YES	YES	NO	YES	YES	6/10
Yilmaz Yelvar et al. (2017) [31]	YES	YES	NO	YES	NO	NO	YES	YES	NO	YES	YES	6/10
Thomas et al. (2016) [37]	YES	YES	YES	YES	NO	NO	NO	YES	NO	YES	YES	6/10
